# Adenoviral VEGF-D^ΔN ΔC^ gene therapy for myocardial ischemia

**DOI:** 10.3389/fbioe.2022.999226

**Published:** 2022-12-22

**Authors:** Juho Pajula, Johanna Lähteenvuo, Markku Lähteenvuo, Krista Honkonen, Paavo Halonen, Olli-Pekka Hätinen, Antti Kuivanen, Minja Heikkilä, Jussi Nurro, Juha Hartikainen, Seppo Ylä-Herttuala

**Affiliations:** ^1^ A.I. Virtanen Institute, University of Eastern Finland, Kuopio, Finland; ^2^ Heart Center and Gene Therapy Unit, Kuopio University Hospital, Kuopio, Finland

**Keywords:** animal models of human disease, gene therapy, angiogenesis, coronary artery disease, VEGF-D

## Abstract

**Background:** Cardiovascular diseases are the leading cause of death globally. In spite of the availability of improved treatments, there is still a large group of chronic ischemia patients who suffer from significant symptoms and disability. Thus, there is a clear need to develop new treatment strategies for these patients. Therapeutic angiogenesis is a novel therapy method which has shown promising results in preclinical studies. In this study, we evaluated safety and efficacy of adenoviral (Ad) VEGF-D^ΔNΔC^ gene transfer for the treatment of myocardial ischemia in a pig model.

**Methods:** Adenoviral VEGF-D^ΔNΔC^ gene transfer was given to pigs (*n* = 26) *via* intramyocardial injections using an electromechanical injection catheter. Angiogenic effects were evaluated in an acute myocardial infarction model (*n* = 18) and functionality of the lymphatic vessels were tested in healthy porcine myocardium (*n* = 8). AdLacZ was used as a control.

**Results:** AdVEGF-D^ΔNΔC^ induced safe and effective myocardial angiogenesis by inducing a four-fold increase in mean capillary area at the edge of the myocardial infarct six days after the gene transfer relative to the control AdLacZ group. The effect was sustained over 21 days after the gene transfer, and there were no signs of vessels regression. AdVEGF-D^ΔNΔC^ also increased perfusion 3.4-fold near the infarct border zone relative to the control as measured by fluorescent microspheres. Ejection fraction was 8.7% higher in the AdVEGF-D^ΔNΔC^ treated group 21 days after the gene transfer relative to the AdLacZ control group. Modified Miles assay detected a transient increase in plasma protein extravasation after the AdVEGF-D^ΔNΔC^ treatment and a mild accumulation of pericardial effusate was observed at d6. However, AdVEGF-D^ΔNΔC^ also induced the growth of functional lymphatic vasculature, and the amount of pericardial fluid and level of vascular permeability had returned to normal by d21.

**Conclusion:** Endovascular intramyocardial AdVEGF-D^ΔNΔC^ gene therapy proved to be safe and effective in the acute porcine myocardial infarction model and provides a new potential treatment option for patients with severe coronary heart disease.

## Introduction

Cardiovascular diseases are the leading cause of mortality and hospitalization in the western world (Naghavi et al., 2017). Coronary angioplasty and stenting are important treatment methods together with coronary artery bypass graft (CABG) surgery to treat severe coronary heart disease. In spite of the availability of the improved treatment methods, there is still a group of patients who suffer from significant symptoms and disability and are not eligible for coronary interventions due to diffuse stenosis, small coronary vessels, repeated operations or too high risk of the operation. Thus, there is an urgent need to develop new treatments for these patients (Giacca and Zacchigna, 2012; Ylä-Herttuala and Baker, 2017). These patients might benefit from catheter mediated gene therapy with angiogenic growth factors.

Myocardial gene therapy is a method that aims to induce local expression of a therapeutic agent by using local delivery of gene transfer vectors (Korpela et al., 2021). The aim is to induce therapeutic angiogenesis, which refers to the stimulation of blood vessel growth and enlargement. This can potentially be utilized in treating coronary heart disease by increasing myocardial perfusion and cardiac function (Ylä-Herttuala et al., 2017).

Therapeutic angiogenesis has shown potential for the treatment of myocardial ischemia (Rissanen et al., 2003a; Lähteenvuo et al., 2009). A local increase in perfusion could be beneficial in the infarct border zone and in the hibernating myocardium near the infarct zone. Fast, efficient induction of angiogenesis could be achieved by adenoviral (Ad) overexpression of angiogenic growth factors, such as VEGF-A or FGF-4 (Ylä-Herttuala and Alitalo, 2003). However, the first attempts to use VEGF-A or FGF-4 in clinical trials have not provided clinically significant results (Giacca and Zacchigna, 2012; Ylä-Herttuala et al., 2017). Therefore, there is a clear need to test other potential factors with more optimal properties for intracardiac therapy.

Vascular endothelial growth factor-D (VEGF-D) is a promising new member of the VEGF-family (Rissanen et al., 2003a), which binds to VEGFR-2 (Flk-1,KDR) and VEGFR-3 (Flt-3) inducing angiogenesis and lymphangiogenesis, respectively (Achen et al., 1998; Marconcini et al., 1999; Rissanen et al., 2003a). It is present in the vasculature as a precursor protein that has a high affinity for VEGFR-3 (7). Proteolytic processing of the VEGF-D precursor protein leads to the removal of the N and C terminal ends (VEGF-D^ΔNΔC^), which increases its affinity to VEGFR-2 and highly improves its angiogenic properties (Baldwin et al., 2001). VEGF-D^ΔNΔC^ has several advantages compared to previously used VEGF-A, such as slower but longer-term signalling kinetics through VEGFR-2 and it diffuses better in the transduced tissues. It also binds to Neuropilin-1 and -2, which improves angiogenic effects (Jauhiainen et al., 2011). Previous preclinical studies have shown promising results *in vitro* (Toivanen et al., 2009)and in skeletal muscle and myocardium (Rissanen et al., 2003a; Toivanen et al., 2009; Leikas et al., 2021). In this study, we evaluated safety and efficacy of AdVEGF-D^ΔNΔC^ for the treatment of myocardial ischemia in porcine myocardium. The study suggests that AdVEGF-D^ΔNΔC^ gene therapy is a safe and effective new option for the treatment of severe myocardial ischemia.

## Materials and methods

### Study overview

Domestic farm pigs were used to study the effects of AdVEGF-D^ΔNΔC^ gene transfer. The study included two sets of animals. In the first set, angiogenic effects were evaluated in ischemic conditions (*n* = 18). Animals were divided into four groups: AdVEGF-D^ΔNΔC^ day 6 (*n* = 6), day 21 (*n* = 3), AdLacZ day 6 (n = 6) and day 21 (*n* = 3). Acute myocardial infarction (AMI) was induced in the anterolateral wall of the left ventricle by catheter mediated occlusion of the distal part of the LAD with a VortX-18 occlusion coil (Boston Scientific, United States) (Lähteenvuo et al., 2009). Total occlusion of the coronary artery was confirmed by angiography after the appearance of ischemic ECG changes and a wall motion defect detected with intracardiac ultrasound imaging. After the gene transfer pigs were followed for 6 and 21 days. In the second set of experiment functionality tests of the lymphatic vessels were done in healthy porcine myocardium (n = 8). Animals were divided into two groups AdVEGF-D^ΔNΔC^ (*n* = 4) and AdLacZ (*n* = 4). After the gene transfer pigs were followed for 6 days. All animal experiments were approved by the Animal Experiment Board in Finland.

### Gene transfer

Pigs received an intramyocardial gene transfer of AdVEGF-D^ΔNΔC^ or marker gene AdLacZ as a control. Ten injections (200 μl each) using a total dose of 1 × 10^12^ vp diluted in sterile saline to 2 ml were given using an 8 F NOGA™ Myostar catheter system (Johnson&Johnson, United States). The ischemic group received gene transfer 30 min after the induction of AMI around the infarct area, the border zone of the movement defect and the normally contracting myocardium proximal to the infarction area where the formation of collateral vessels was desired.

### Echocardiography

Echocardiography was performed at the baseline, after AMI and before sacrifice with an Acuson Acunav ultrasound Catheter (Siemens Medical Solutions, United States) inserted into the right atrium *via* the femoral vein. Longitudinal 2-chamber views are shown to visualize pericardial effusion six days after the gene transfer. The ejection fraction was quantified using a modified Simpson’s method.

### Lymphangiography and lymphatic vessel functionality

Lymphatic vessels were visualized with *in vivo* lymphangiography using Innova 3100IQ (GE Healthcare, United States). Gadolinium contrast agent was injected into the myocardium under echo guidance. Lymphatic vessel functionality was assessed by injecting fluorescent tracers into the myocardium (FITC dextran) by using the NOGA Myostar needle catheter and into the pericardial sac (rhodamine-labeled lectin) by using transthoracic echo guidance.

### Tissue samples

The pigs were sacrificed by injection of saturated magnesium sulfate (MgSO_4_) solution until the detection of cardiac arrest. The hearts were removed and fixed with citric acid-buffered 1% paraformaldehyde (PFA) solution for sampling. In the ischemic group, samples were collected from the maximal transduction area (next to the needle tracts), infarct border zone, infarct scar, interventricular septum (control) and apex (control) for histology (Lähteenvuo et al., 2009). For lymphatic vessel analyses, myocardium samples were collected from maximal transduction area, posterior wall (control) and also mediastinal lymph nodes were collected.

### Evans blue measurement for vascular permeability

Vascular permeability was determined by leakage of Evans blue-labeled plasma proteins. Evans blue dye (Sigma-Aldrich, 30 mg/kg) was injected intravenously 30 min before sacrifice. The heart was perfusion fixed with 1% PFA in 0.05 M citrate buffer. Samples were collected from the maximal transduction area, infarct border zone, infarct scar and control samples from the apex and interventricular septum. The dye was extracted to formamide by incubating the samples for 48 h in +60°C. Evans blue absorbance was measured with a spectrophotometer at 610 nm and ratios between transduced and control areas were calculated.

### Microsphere measurement for tissue perfusion

Perfusion in the transduced areas was measured using fluorescent microsphere particles (Rissanen et al., 2003b). Microsphere particles (Molecular Probes, diameter 15 μm, 5 × 10^6^ particles in 10 ml) were injected into the left ventricle near the mitral valve. Samples were collected from the maximal transduction area, infarct border zone, infarct scar and control samples from the apex and interventricular septum. The samples were dissolved in ethanol-KOH and the microspheres were isolated according to the manufacturer’s instructions. The number of microspheres was quantified with a spectrophotometer and the ratio between transduced and control areas was calculated.

### Histology

Immunostainings were performed on 7 μm thick PFA fixed paraffin embedded tissue sections using avidin-biotin-HRP and alkaline-phosphatase systems with 3′-5′-diaminobenzidine color substrate (Vector Laboratories Inc.) (Lähteenvuo et al., 2009). *ß* -Galactosidase (Z378B, Promega) immunostaining was used to analyse NOGA Myostar catheter transduction efficiency. For capillary vessel analyses PECAM-1 (AB28364, Abcam) immunostaining was done. To analyze pericyte coverage of the vessels α-SMA (M0851, Dako) immunostaining was done. Lymphatic vessels were identified by LYVE-1 (AF 2089, R&D systems) immunostaining. GAP43 (AB5220, Millipore) and tyrosine hydroxylase (AB1542, Millipore) immunostainings were used to analyze cardiac nerves (Lähteenvuo et al., 2020). Immune infiltration in the heart was evaluated by using CD-3 staining (MA1-90582, Thermo Fisher scientific). For major tissues histopathology H&E stainings (Hematoxylin and Eosin) were performed. Standardized protocols and non-immune control samples were used (Lähteenvuo et al., 2009).

### Blood vessel measurements

The mean capillary area (μm^2^) was measured from PECAM-1 immunostained myocardial sections. Samples were collected from the infarct scar area, infarct border zone and the maximal transduction area and from the healthy myocardium above the infarction scar. All measurements were performed in a blinded manner from 5 different randomly selected fields from each muscle section. The total capillary area was calculated as a ratio of the image area (Rissanen et al., 2003a).

### Safety analyses

Off-target tissue samples were collected from lung, liver, spleen kidney and ovario. Blood samples were collected and tested 21 days after the gene transfer. Alkaline phosphatase (ALP) and alanine aminotransferase (ALT) were used to analyze the liver function and Creatinine (Creat) for kidney function. For potential tissue damage lactate dehydrogenase (LD) and heart muscle-specific marker troponin I (TnI) were tested. Also, inflammation marker C-reactive protein (CRP) was analyzed in a veterinary laboratory service (Movet Oy, Kuopio, Finland). For cardiac nerve analyses the second set of animals were used (*n* = 8) for GAP43 and tyrosine hydroxylase immunostainings. Nerve endings were calculated from 5 randomly selected fields.

### Statistics

All *p*-values were calculated using a two-tailed student’s t-test after ANOVA analysis. All values are indicated as mean +SEM. A *p*-value of <0.05 (marked * in the figures) was considered statistically significant. Statistical analyses and graphs were done with GraphPad Prism program (GraphPad Software, United States).

## Results

### NOGA gene transfer efficiency

**FIGURE 1:**
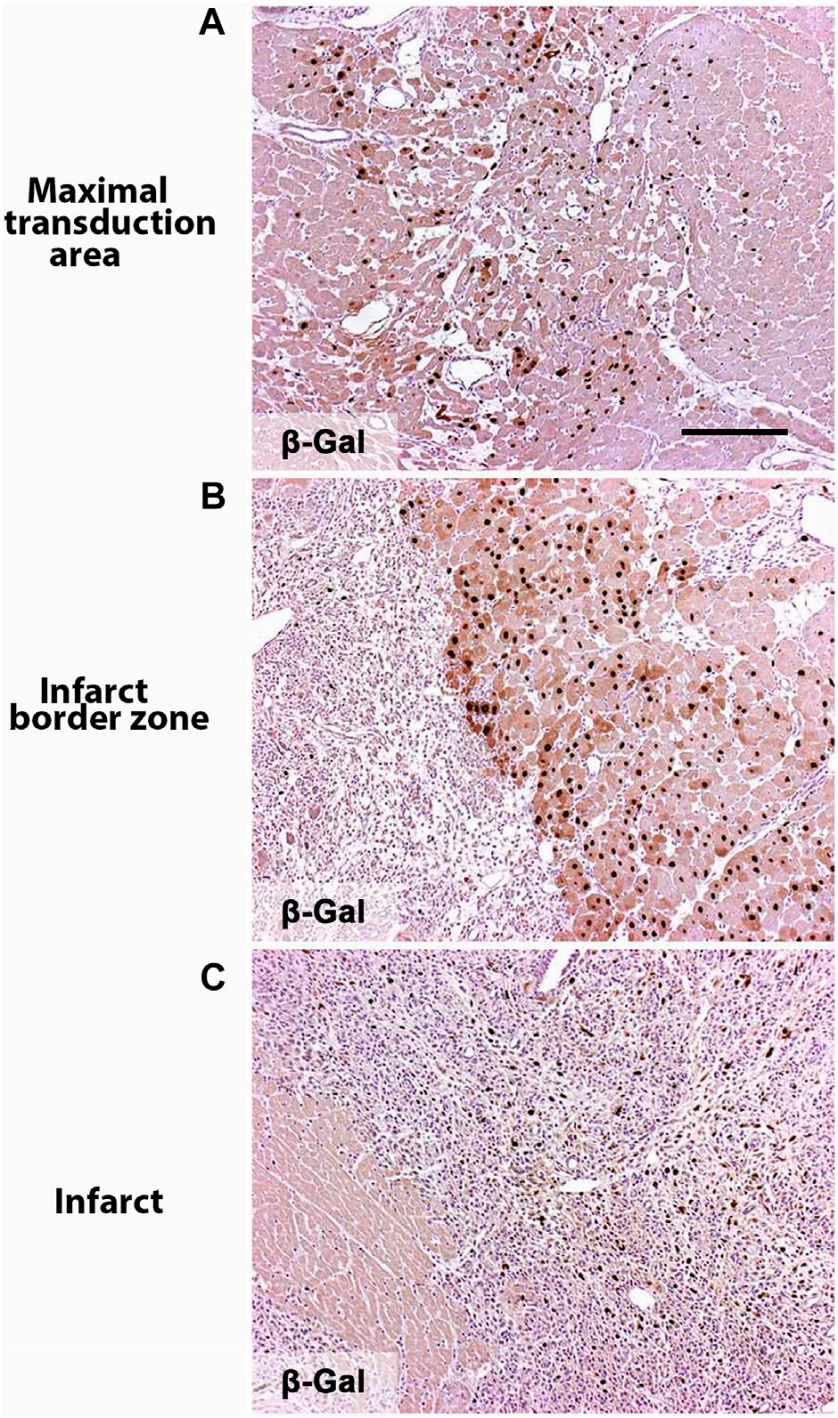
Noga gene transfer efficiency. Nuclear targeted *ß*-galactosidase expression in the heart showing AdLacZ transduced nuclein in brown 6 days after the gene transfer. **(A)** Maximal transduction area is collected next to the needle tract. **(B)** Infarct border zone. **(C)** Infarct zone. Magnification ×100. Scale bar 100 µm.

Each intramyocardial injection produced a transduction area of about 1 cm^3^. The approximated gene transfer efficiency in the immediate proximity of the needle track was over 50%. Very few *ß*-galactosidase positive cells were found in the infarct scar, but 10%–20% of the cells within the fibroblast and inflammatory cell-rich border zone were positive (Figure 1). No *ß*-galactosidase positive cells were found 21 days after the gene transfer since adenoviral gene expression in large animals lasts approximately two weeks (Rissanen et al., 2003a) (data not shown).

### AdVEGF-D^ΔNΔC^ induces angiogenesis

**FIGURE 2:**
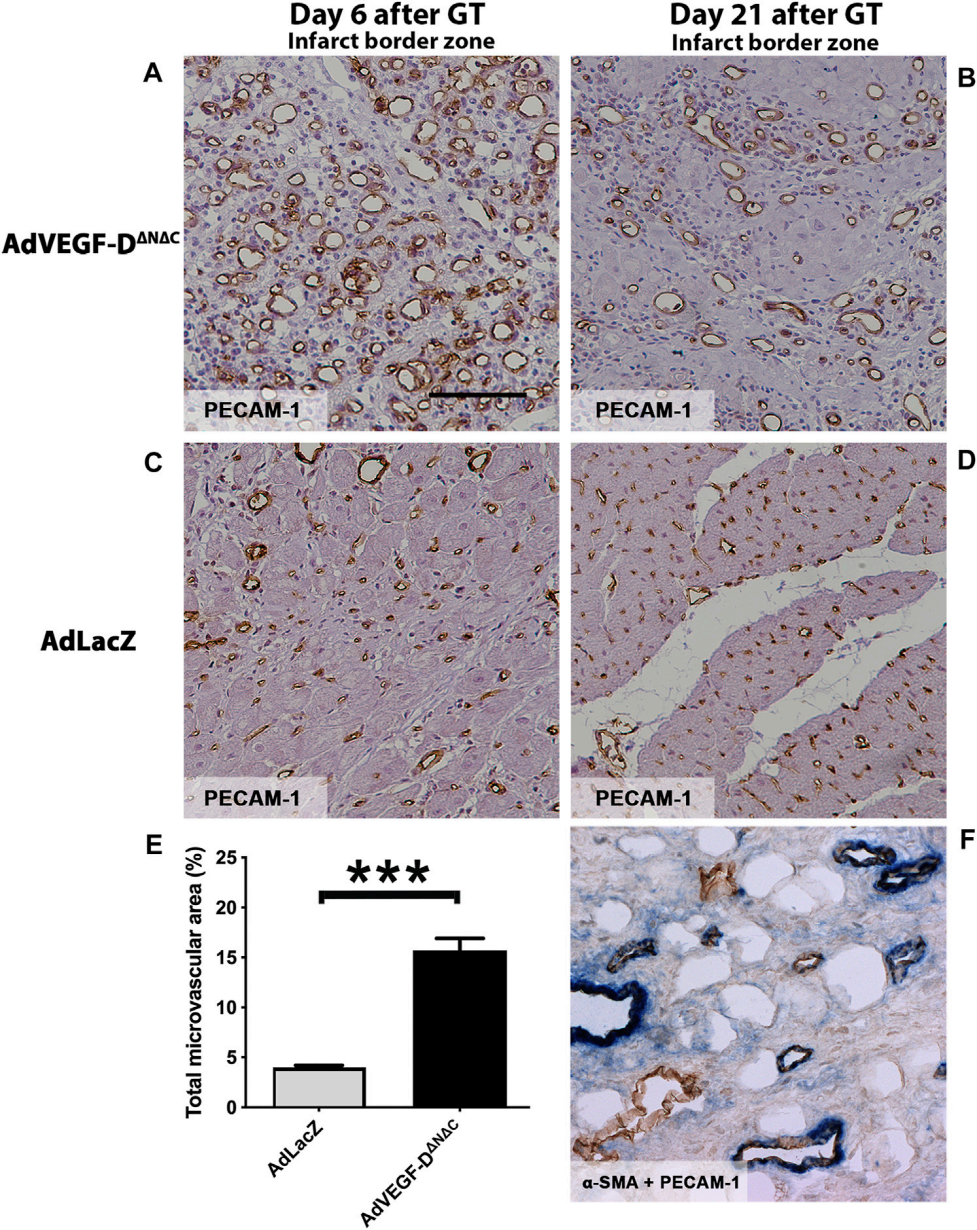
AdVEGF-DΔNΔC induces angiogenesis in the ischemic porcine heart. **(A)** AdVEGF-D^ΔNΔC^ induced robust angiogenic effect 6 days after the gene transfer (GT). **(B)** 21 days after the AdVEGF-D^ΔNΔC^ gene transfer the angiogenic effect was still present. **(C,D)** AdLacZ gene transfer did not induce angiogenesis. **(E)** The graph shows the total microvascular area (%) on day 6. The difference between the groups was statistically significant (*p* < 0.001). **(F)** Double immunostaining α-SMA + PECAM-1 to show pericytes around the vessels on day 21 in AdVEGF-D^ΔNΔC^ group. PECAM-1 - brown color, α-SMA–blue color. PECAM-1 staining for infarct border zone samples, magnification ×200, scale bar 100 µm.

Six days after the gene transfer AdVEGF-D^ΔNΔC^ had induced a clear angiogenic effect (Figure 2A). The growth of capillaries was observed in all muscle layers, with the maximal effect in the epicardium. AdLacZ did not alter capillary size or morphology (Figure 2C). The mean capillary area in the AdVEGF-D^ΔNΔC^ group was 4-fold (15.7% of the total area) relative to the AdLacZ group (4.0% of the total area) six days after the gene transfer in the infarct border zone (*p* < 0.0001) (Figure 2E). Three weeks after the gene transfer the enlarged capillaries and increased number of small arterioles were still present in the AdVEGF-D^ΔNΔC^-transduced hearts (11.2% of the total area) (Figures 2B,F). Some endogenous angiogenesis was observed in the infarct border zone in the AdLacZ group (Figure 2C). Very few vessels were observed in the scar area. Thus, the AdVEGF-D^ΔNΔC^ gene transfer produced a robust angiogenic response in the infarct border zone.

### AdVEGF-D^ΔNΔC^ gene transfer induces the growth of functional lymphatic vessels

**FIGURE 3:**
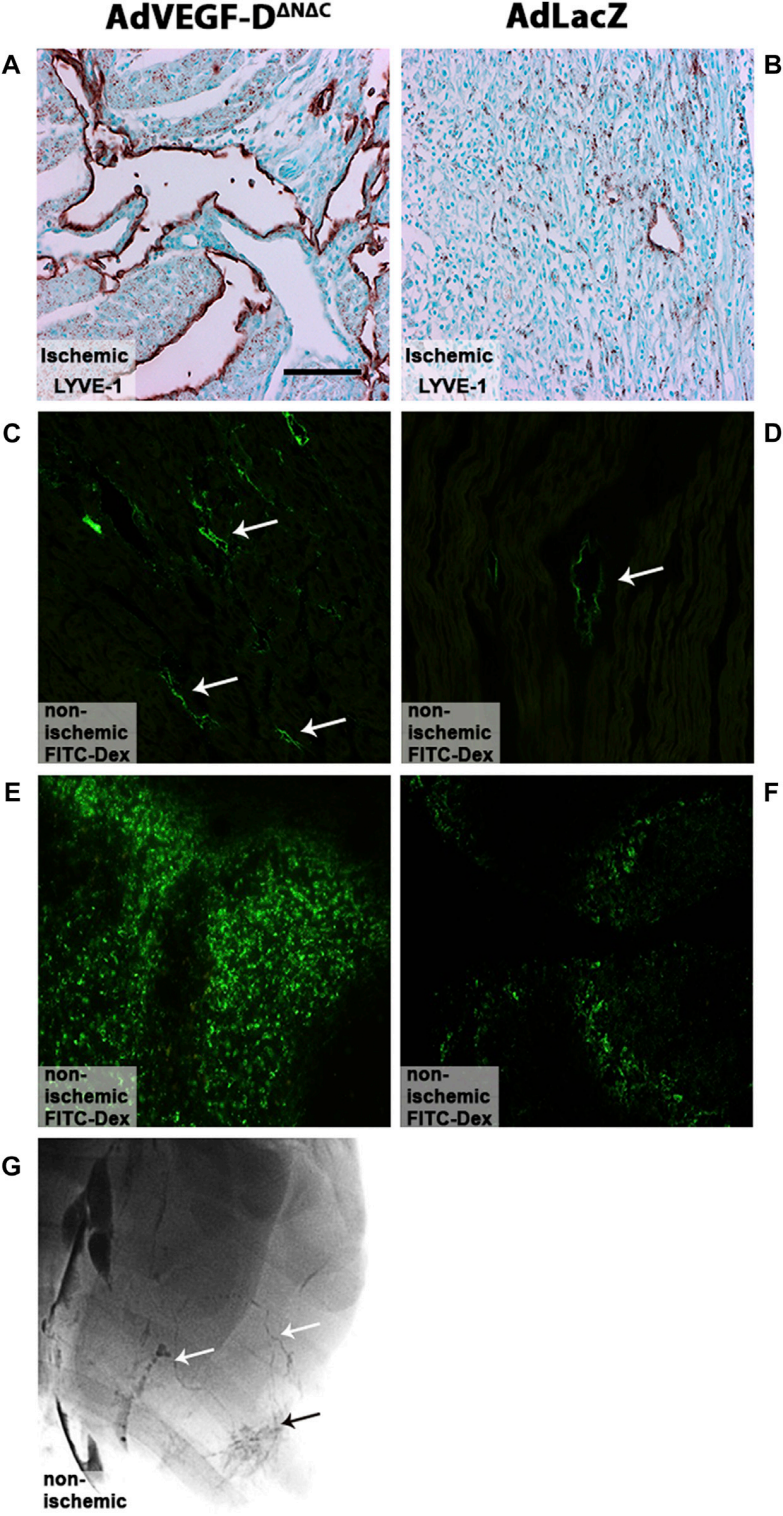
AdVEGF-D^ΔNΔC^ gene transfer induces lymphangiogenesis. **(A)** AdVEGF-D^ΔNΔC^ induced lymphangiogenesis. **(B)** AdLacZ did not affect lymphatic vessels. **(C,D)** FITC labeled fluorescent dextran was taken up by the lymphatics in both groups (arrows). **(E,F)** 30 min after injection large quantities of the tracer were also found in mediastinal lymph nodes in both groups. **(G)** Draining lymphatic vessels (white arrows) and dense lymphatic network (black arrow) in the gene transfer area were visualized with lymphangiography in AdVEGF-D^ΔNΔC^ group. LYVE-1 staining **(A,B)**, Representative images, maximal trasduction area. Magnification ×200. Scale bar 100 µm.

LYVE-1 positive lymph vessel sprouting was observed in the AdVEGF-D^ΔNΔC^ gene transfer area (Figure 3A). In the AdLacZ control group, only a few lymphatic vessels were detected (Figure 3B). Injected FITC labeled fluorescent dextran was efficiently taken up by the lymphatics in the gene transfer area in both groups (Figures 3C,D). 30 min after the injection large quantities of the tracer were also found in the mediastinal lymph nodes in both groups (Figures 3E,F). Both dense lymphatic network in the gene transfer area and draining lymphatic vessels were observed in lymphangiography (Figure 3G).

### AdVEGF-D^ΔNΔC^ increases vascular permeability

**FIGURE 4:**
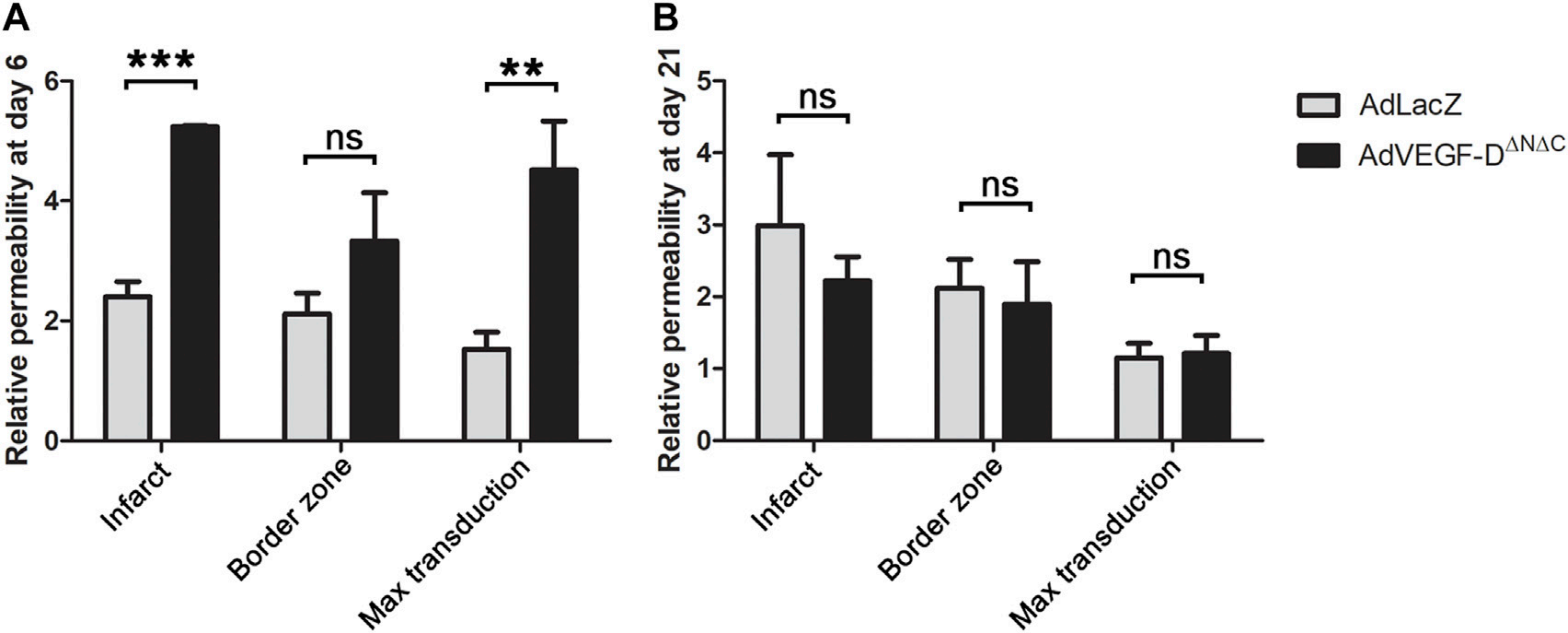
AdVEGF-D^ΔNΔC^ increases vascular permeability at day 6. **(A)** AdVEGF-D^ΔNΔC^ increased vascular permeability as compared to AdLacZ at day 6. **(B)** At day 21 there were no statistically significant differences between the groups. ns = non-significant (*p* > 0.05). ** = *p* < 0.01. *** = *p* < 0.001. Figures shows mean permeability ratios to control area with standard errors.

AdVEGF-D^ΔNΔC^ increased permeability approximately 2.2-fold as compared to AdLacZ in all transduced areas (permeability ratios: infarct area 2.18 (*p* = 0.0002), edge 1.58 (*p* = ns) and maximal transduction area 2.95 (*p* = 0.0011). Twenty-one days after the intramyocardial injections the vascular permeability ratios of AdVEGF-D^ΔNΔC^ transduced hearts had returned to the level of AdLacZ controls (*p* = ns) (Figure 4). The amount of pericardial effusion was semi-quantitatively analyzed from longitudinal ultrasound images at the time of sacrifice on day 6 and 21 after the gene transfer. In the AdVEGF-D^ΔNΔC^ group, every animal had moderate amount of pericardial effusion on day 6 and a small amount of effusion on day 21. No pericardial effusion was seen in the AdLacZ group. There were no deaths caused by cardiac tamponade.

### AdVEGF-D^ΔNΔC^ gene transfer improves cardiac function

**FIGURE 5:**
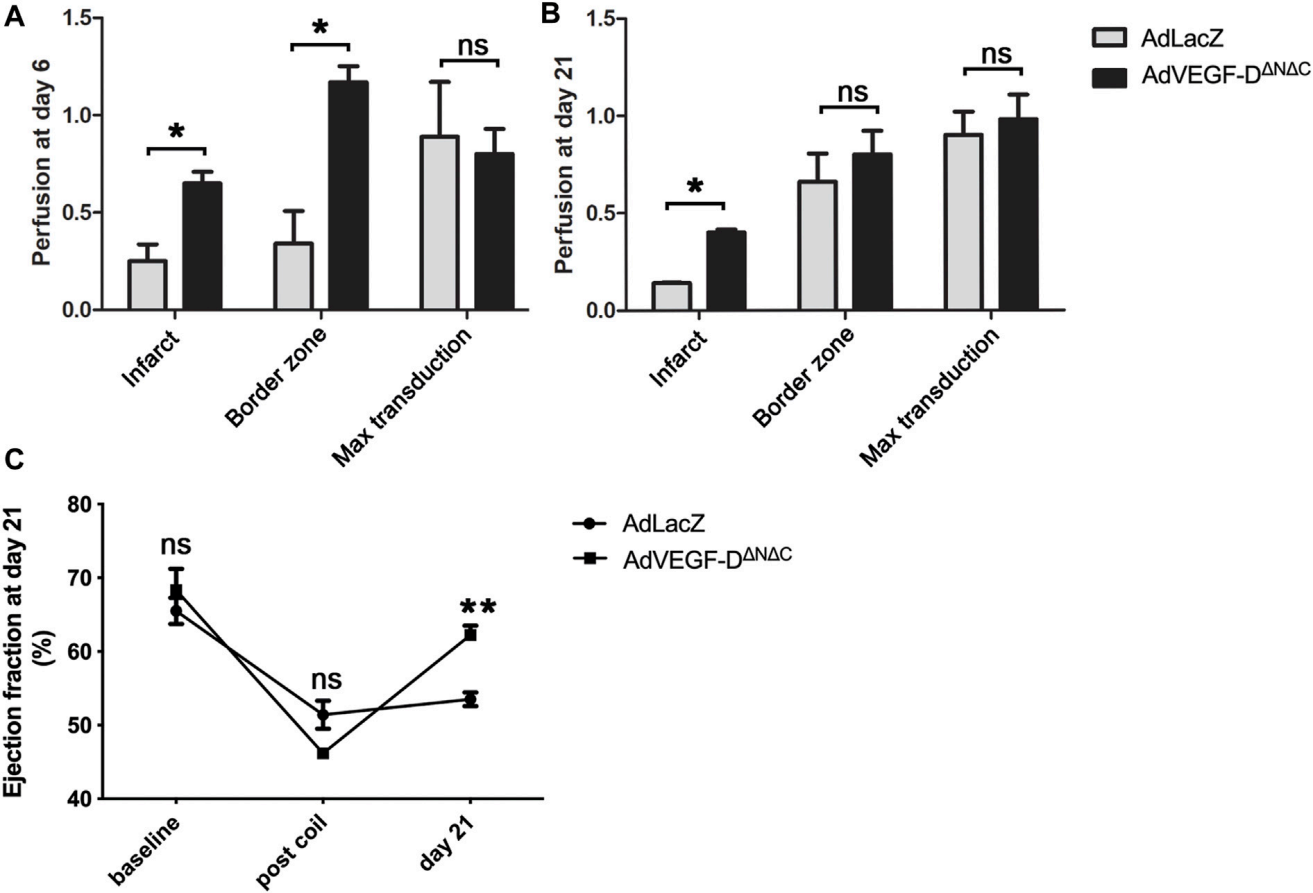
AdVEGF-D^ΔNΔC^ increases perfusion and ejection fraction. **(A)** At day 6 AdVEGF-D^ΔNΔC^ increased perfusion in the infarct and in the infarct border zone compared to AdLacZ. **(B)** At day 21 slight increase in perfusion was still observed. **(C)** At day 21 echocardiography showed a statistically significant increase in ejection fraction in the AdVEGF-D^ΔNΔC^ group. ns = non-significant (*p* > 0.05). * = *p* < 0.05. ** = *p* < 0.01. Figures A and B show mean perfusion ratios to control area with standard errors.

AdVEGF-D^ΔNΔC^ induced a 3.4-fold increase in perfusion in the infarct border zone and a 2.6-fold increase in the infarction area relative to AdLacZ. Increase in perfusion was still observed 21 days after the gene transfer in the infarct area (Figures 5A,B).

Echocardiography showed an increase of 16% in the ejection fraction 21 days after the gene transfer compared to the time of occlusion (*p* = 0.0002), whereas AdLacZ induced a non-significant change of 2% (*p* = ns). There were no differences between the groups in the ejection fraction at baseline and after the occlusion of the vessel (*p* = ns) (Figure 5C).

### AdVEGF-D^ΔNΔC^ had no effects on measured safety parameters

**FIGURE 6:**
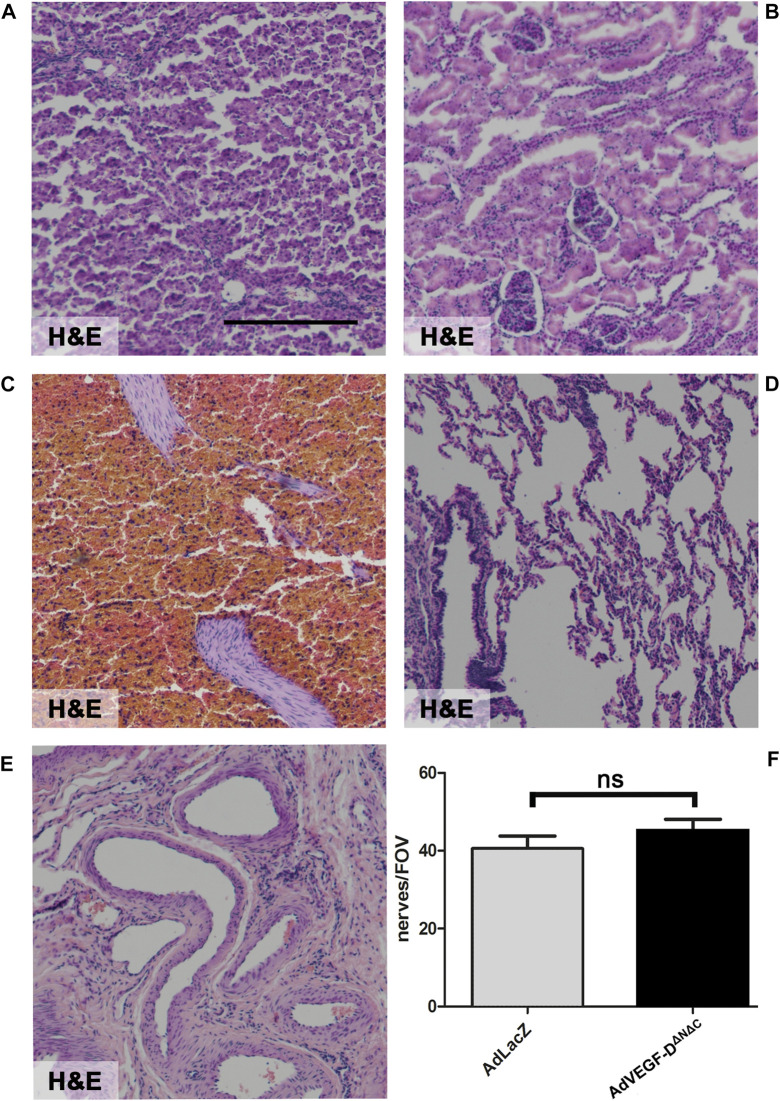
AdVEGF-D^ΔNΔC^ had no effect on off-target tissues or cardiac nerves. Gene transfer of AdVEGF-D^ΔNΔC^ did not affect off-target tissue histology. **(A)** Liver **(B)** Kidney **(C)** Spleen **(D)** Lung **(E)** Ovario. **(F)** The graph shows nerve endings per field of view. The difference between the groups was not statistically significant. ns = non-significant (*p* > 0.05). Hematoxylin and Eosin staining **(A–E)**. ×40 Magnification. Scale bar 100 µm.

There were no inflammatory or histological changes in the collected off-target tissues (lung, liver, spleen, kidney, and ovary) (Figures 6A–E). There were no significant changes in tested blood parameters 21 days after the gene transfer (*n* = 3) (Table 1).

**TABLE 1:** AdVEGF-D^ΔNΔC^ gene transfer did not cause significant changes in blood parameters. Table shows mean values and standard deviations before the gene transfer and 21 days after the gene transfer.

	Before GT	21 days after GT
ALP U/l	104.0 ± 12.2	80.9 ± 11.1
ALT U/l	25.4 ± 6.0	23.2 ± 4.9
CRP mg/l	4.7 ± 2.1	2.5 ± 1.3
Creat umol/l	118.1 ± 13.0	142.5 ± 10.7
LD U/l	224.7 ± 53.4	203.0 ± 10.4
TnI ng/ml	<0.2 ± 0.00	<0.2 ± 0.00

ALP, alkaline phosphatase; ALT, alanine aminotransferase; CRP, C-reactive protein; Creat, creatinine; LD, lactate dehydrogenase; TnI, troponin I. GT = Gene transfer.

**FIGURE 7:**
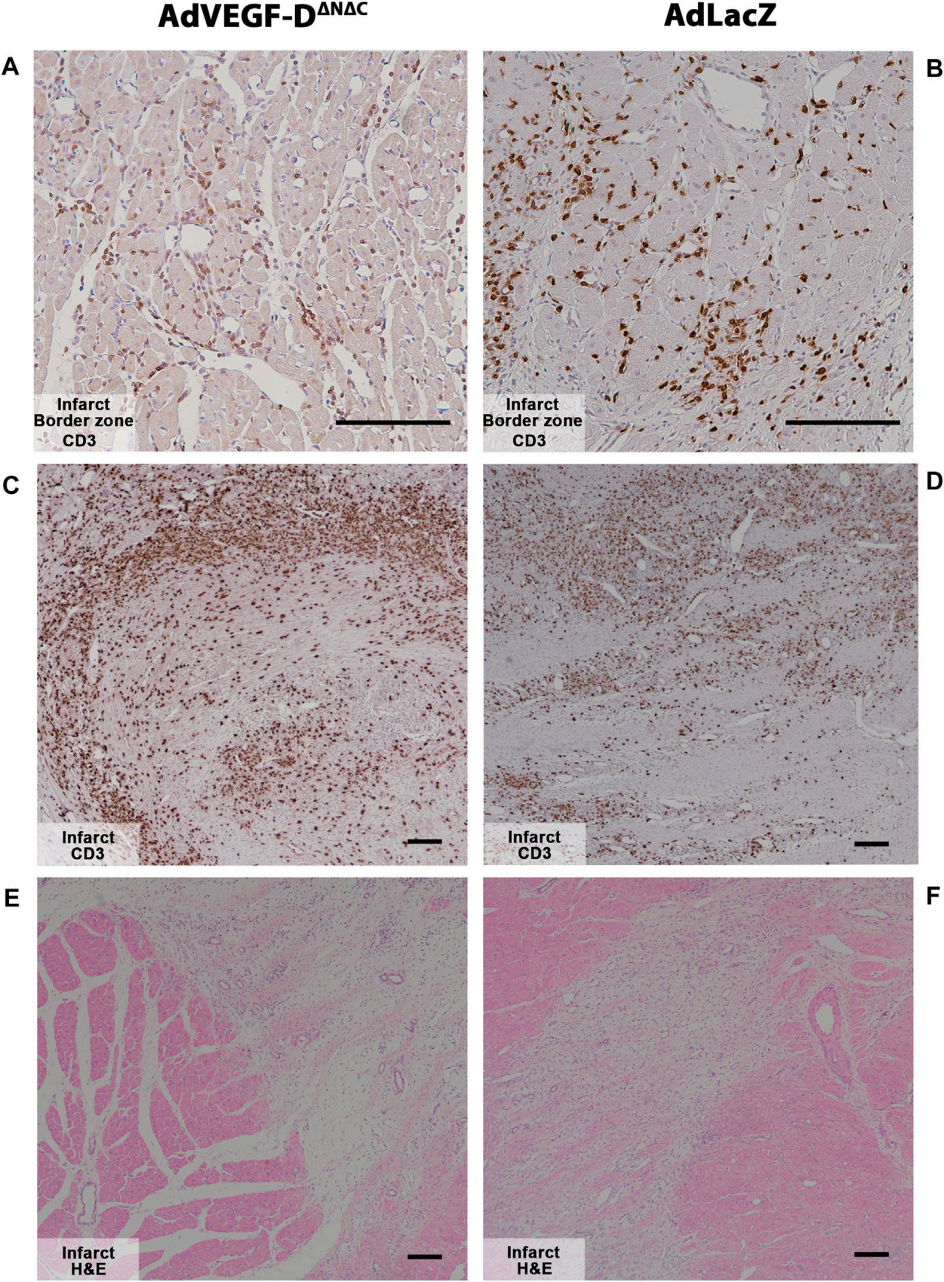
Myocardial infarction causes strong inflammatory responses in both groups at day 6. **(A,B)** AdVEGF-D^ΔNΔC^ and AdLacZ had no differences in the inflammatory response in the infarct border zone. Magnification ×200. **(C–F)** Myocardial infarct caused strong inflammatory responses in both groups. Magnification ×40. CD3—brown. Representative images. Scale bar 100 µm.

Inflammatory cell responses were analyzed semi-quantitatively from the ischemic hearts. There were no differences compared to AdLacZ group. Both groups had strong immune infiltration in the infarct area (Figure 7).

Gene transfer of AdVEGF-D^ΔNΔC^ did not affect cardiac nerves. There were no statistically significant differences in nerve density and no signs of increased nerve branching or sprouting compared to the AdLacZ group (Figure 6F).

## Discussion

Percutaneous NOGA Myostar catheter delivery to the ischemic area in the infarcted heart was feasible and did not cause any major safety concerns. AdVEGF-D^ΔNΔC^ induced the growth of both angiogenic and lymphangiogenic vessels and it also led to an increase in blood capillary area at the infarct border zone six days after the gene transfer. The increase in blood capillary area was still measurable 21 days after the gene transfer. Since the adenoviral gene expression only lasts for around two weeks, and non-functional vessels have been shown to regress quickly (Rissanen et al., 2003a), it is likely that the effects on microvascular area and perfusion were stabilized by hemodynamics and might be long-lasting. AdVEGF-D^ΔNΔC^ led to an increase in perfusion six days after the gene transfer and this increase, although to a lesser extent, was still seen 21 days after the gene transfer. Increased perfusion in the infarction edge area seems to increase perfusion also in the infarct area. Since VEGF-D^ΔNΔC^ does not bind to heparan sulphate proteoglycans, it is easily diffusible in heart tissues thus extending its biological effects to wider areas in myocardium than other members of the VEGF family (Jauhiainen et al., 2011). The perfusion measured with microspheres was slightly lower in the maximal transduction area of AdVEGF-D^ΔNΔC^ transduced animals as compared to AdLacZ. This may be due to the fact that the microspheres were only 15 μm in diameter. In maximally transduced area mean capillary diameter exceeded the size of the particles and some of them may have been washed away from the tissue.

AdVEGF-D^ΔNΔC^ gene transfer also led to an increase in ejection fraction 21 days after the infarction. A transient increase in permeability was found in the treated areas at day 6 in the AdVEGF-D^ΔNΔC^ group, but this had returned to normal by day 21. It is possible that the decrease in permeability 21 days after the gene transfer in the presence of increased perfusion indicates vessel maturation. However, lymphangiogenic vessel growth may also contribute to the reduction of plasma protein extravasation. In this study, we showed that AdVEGF-D^ΔNΔC^ gene transfer induces the growth of functional lymphatic vessels that integrated into the existing lymphatic vasculature. This could potentially reduce the risk of cardiac edema and tamponade since excess fluid escaping into the pericardial sac due to angiogenic leaky vessels could be removed *via* lymph drainage. This is an important safety issue since it has been shown previously that classical VEGF-A gene transfer greatly increases vessel permeability and possesses a clear safety concern to the patients (Ylä-Herttuala et al., 2017; Korpela et al., 2021). To analyze lymphangiogenesis we decided to use healthy pig hearts to exclude changes caused by infarction thus giving us proof of the AdVEGF-D^ΔNΔC^ lymphangiogenic effects.

For safety reasons we evaluated cardiac nerve density because it has been previously shown that AdVEGF-B_186_ gene transfer caused an increase in the cardiac nerve density which can lead to ventricular arrhythmias (Lähteenvuo et al., 2020). AdVEGF-D^ΔNΔC^ did not affect the number of cardiac nerves compared to the control group. We also checked histology of the off-target tissues and analyzed clinical chemistry from blood samples. We did not detect any safety concerns in the analyzed samples. AdLacZ was used as a control group. This ensures that both groups got the same dose of the adenovirus in order to control for possible adverse effects due to the vector e.g. inflammatory reactions between the study groups.

In conclusion, AdVEGF-D^ΔNΔC^ is a promising new therapeutic candidate for severe coronary heart disease, as it induced angiogenesis, increased perfusion and improved cardiac function as well as increased the growth of functional lymphatic network. AdVEGF-D^ΔNΔC^ has also entered clinical testing (Hartikainen et al., 2017; Korpela et al., 2021; Leikas et al., 2022). However further studies are needed to optimize the beneficial effects, evaluate the long-term safety and find the patient groups that may benefit from the treatment.

## Data Availability

The raw data supporting the conclusion of this article will be made available by the authors, without undue reservation.
